# Deciphering Sequence Determinants of Zygotic Genome Activation Genes: Insights From Machine Learning and the ZGAExplorer Platform

**DOI:** 10.1111/cpr.70039

**Published:** 2025-04-18

**Authors:** Jixiang Xing, Siqi Yang, Yuchao Liang, Pengwei Hu, Bingjie Dai, Hanshuang Li, Yongqiang Xing, Yongchun Zuo

**Affiliations:** ^1^ State Key Laboratory of Reproductive Regulation and Breeding of Grassland Livestock, Institutes of Biomedical Sciences, School of Life Sciences Inner Mongolia University Hohhot China; ^2^ School of Life Science and Technology Inner Mongolia University of Science and Technology Baotou China

**Keywords:** epigenetic modification, gene conservation, machine learning, sequence specificity, zygotic genome activation

## Abstract

The mammalian life cycle initiates with the transition of genetic control from the maternal to the embryonic genome during zygotic genome activation (ZGA), which becomes pivotal for development. Nevertheless, understanding the conservation of genes and transcription factors (TFs) that underlie mammalian ZGA remains limited. Here, we compiled a comprehensive set of ZGA genes from mice, humans, pigs, bovines and goats, including *Nr5a2* and *TPRX1/2*. The identification of 111 homologous genes through comparative analyses was followed by the discovery of a conserved genetic coding region, suggesting potential sequence preferences for ZGA genes. Notably, an interpretable machine learning model based on *k*‐mer core features showed excellent performance in predicting ZGA genes (area under the ROC curve [AUC] > 0.81), revealing abundant and intricate 6‐base sequence specific patterns and potential binding TFs, including motifs from NR5A2 and TPRX1/2. Further analysis demonstrated that gene sequence features and epigenetic modification features play equally important roles in regulating ZGA genes. Ultimately, we developed the ZGAExplorer platform to provide an invaluable resource for screening ZGA genes. Our study unravels the sequence determinants of ZGA genes across species through multi‐omics data integration and machine learning, yielding insights into ZGA regulatory mechanisms and embryonic developmental arrest.

## Graphical abstract

We compiled a comprehensive set of ZGA genes from mice, humans, pigs, bovines and goats, demonstrating the cross‐species conservation. An interpretable machine learning model, based on *k*‐mer core features, accurately predicted ZGA genes, revealing abundant and intricate six‐base sequence‐specific patterns and potential binding TFs. Additionally, we developed ZGAExplorer, the most comprehensive and user‐friendly ZGA gene information platform to date, providing an invaluable resource for screening ZGA genes.

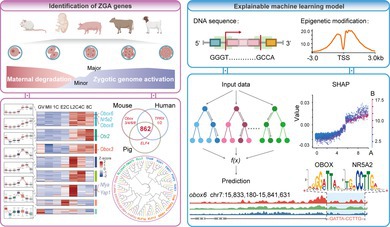

## 1 Introduction

The genome of the mammalian embryo is initially transcriptionally silenced, and preimplantation embryonic development relies on maternal RNA and protein [1, 2, 3, 4]. During the maternal to zygotic transition (MZT), coordinating with the degradation of maternal products, zygotic or embryonic genome activation is initiated (ZGA/EGA, hereafter referred to as just ZGA) [3, 5, 6, 7, 8, 9]. Due to the diversity in developmental strategies employed by different animals, the timing and progression of ZGA vary across species [7, 10]. Compared to fast‐developing *Drosophila* and *Zebrafish* embryos, ZGA occurs slightly later in mammals, which have a more prolonged development [3, 10, 11].

The ZGA process initiates a series of tightly regulated molecular events critical for the progression of embryogenesis, during which both nascent transcripts and the chromatin landscape undergo dramatic changes [7, 12]. However, the essential transcription factors (TFs) that trigger ZGA remain largely unidentified. The discovered TFs associated with ZGA initiation display limited conservation across species, yet the motifs for specific targeting and binding may have some similarities [7, 13, 14, 15, 16, 17, 18]. Many researchers have believed that the motifs of TFs regulating ZGA may enrich in the cis‐regulatory regions of the ZGA genes [4, 13]. It has been reported that a consensus sequence harbouring six motifs corresponding to a subtype of SINE B1/Alu retrotransposon elements enriched the *cis*‐regulatory regions of many mouse ZGA genes, and the Nr5a2 motif is included in it. Experiments have shown that Nr5a2 can activate up to 72% of major ZGA genes [13]. OBOX, a PRD‐like homeobox domain TF family (OBOX1–OBOX8), has also been identified as a key regulator of mouse ZGA. In late two‐cell embryos, ZGA genes contained more OBOX‐binding motifs (TAATCC) at promoters than those specifically activated at other stages [14]. This TF binding motif was also enriched during ZGA in humans and pigs. TPRX1/2/L are critical regulators for ZGA and embryonic development in humans, and their motifs (TAATCC) are enriched in distal open chromatin regions near activated genes upon ZGA [16]. There is evidence that LEUTX may serve as a conserved transcriptional activator, initiating ZGA in early embryos from primates and pigs, and TAATCC constitutes the core component of its motif [18]. The above results indicate that despite species‐specific differences in hitherto reported ZGA regulatory factors, the mechanisms triggering ZGA are evolutionarily conserved from *Drosophila* to mice and possibly to pigs, and the bound motif may also be conserved [19].

During preimplantation embryonic development, histone modification dynamics also exhibit great plasticity and species specificity [20, 21, 22, 23, 24, 25, 26, 27]. The deposition of histone modifications on promoters or enhancers strongly correlates with the expression of ZGA genes [20]. It has been found that broad H3K4me3 reshapes into sharp peaks during ZGA in many mammals [20, 23]. De novo deposition of H3K27ac and H3K4me3 and gradual erasure of H3K27me3 and H3K9me3 are also common phenomena during human and mouse ZGA [10]. Thus, it is necessary to investigate the epigenetic reprogramming specificities orchestrated with ZGA in different mammals.

Currently, machine learning algorithms have garnered extensive application within the realm of biology, particularly in the fields of genomics and epigenetics [28, 29, 30, 31, 32, 33]. For example, Benveniste et al. used the *k*‐mer features of the DNA sequence in the promoter region to construct a logistic regression (LR) classifier that can accurately predict histone modification patterns [34]. In addition, Colbran and colleagues constructed a *k*‐mer‐support vector machine (SVM) machine learning framework and found that the occurrence patterns of short sequence motifs can correctly predict widely active human enhancers [32, 35]. The information encoded in the DNA sequence is pivotal for initiating the processes involving sequence‐specific TFs and histone modifications. Machine learning‐based methods have shown great promise in predicting histone modification patterns and regulatory elements from short DNA motifs [28, 36]. Such success inspires us to explore the sequence‐dependent determinants of mammalian ZGA genes using a computational framework [37, 38, 39, 40].

In this study, we analysed the transcriptional patterns of preimplantation embryos in mice, humans, pigs, bovines and goats, identified ZGA genes in each species, and compiled a comprehensive ZGA gene set. Utilising gene and protein evolutionary analyses, we explored conserved regions in homologous ZGA genes. Additionally, we integrated machine learning algorithms to characterise the sequence preferences and epigenetic modification features of ZGA genes. Finally, we developed ZGAExplorer, a ZGA genes information platform, providing a valuable resource for ZGA gene screening.

## 2 Results

### 2.1 A Comprehensive Set of ZGA Genes Identified From Five Mammals

**FIGURE 1:**
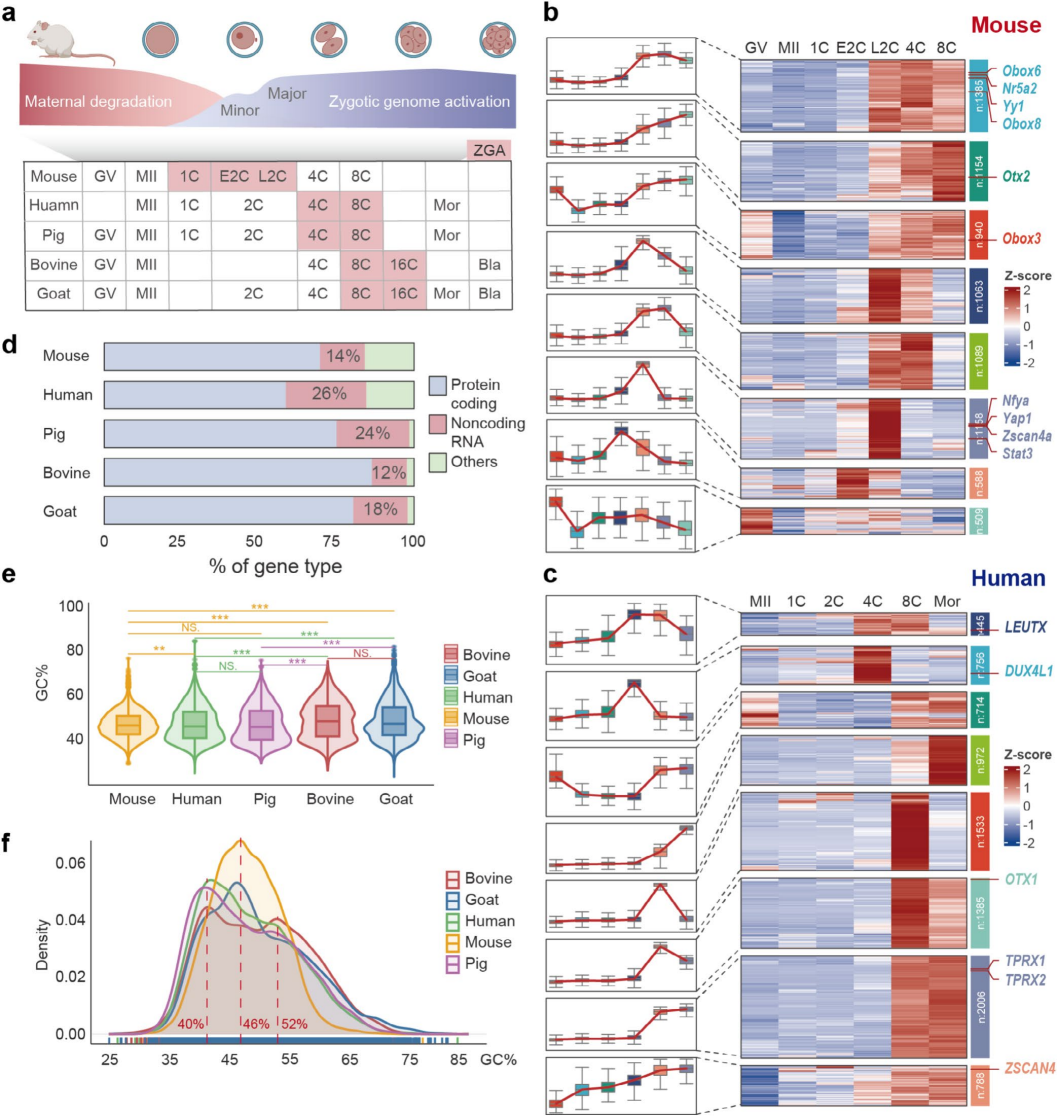
Identification of ZGA candidate genes during mammalian preimplantation embryogenesis. (a) Species and stages that collected RNA‐seq data covered. Red: The period of ZGA occurrence. (b, c) Heatmaps showing transcription levels and dynamics of ZGA genes in fertilised mouse and human embryos. These genes were classified into eight clusters (Clusters 1–8) based on their expression patterns. Representative genes in each cluster are shown. (d) Bar plot showing the proportion of gene types among the ZGA genes of various species. (e) Violin plot depicting the percentage of GC content within the ZGA genes. (f) The density plot of GC percentage for the ZGA genes of distinct species.

ZGA, a pivotal event in successful embryo development, necessitates the comprehensive and precise identification of ZGA genes. This step is crucial for understanding and comparing the dynamics of zygotic genome activation across species [13, 14, 18, 41, 42]. Therefore, we systematically analysed RNA‐seq data from mice, humans, pigs, bovines and goats, encompassing both MII oocytes and various stages of preimplantation embryos (Figure 1a). Previous studies have demonstrated that the identified ZGA gene sets can vary substantially depending on the datasets and identification methods employed. To ensure the comprehensiveness and accuracy of the final ZGA gene set for each species, we referred to the identification methods and candidate ZGA genes from previous studies and incorporated them into our analysis [13, 14, 16, 18].

Overall, we identified 7886 ZGA genes in mice, 8599 in humans, 7927 in pigs, 4900 in bovines and 5419 in goats (Figure 1b,c and Figure S1a; Table S1). The well‐characterised ZGA pivotal genes were also included, such as *Nfya* and *Nr5a2* in mice [13, 43], and *TPRX1/2* and *Dux4* in humans [16, 44, 45]. Intriguingly, we found eight defined clusters of specific binding patterns. Genes in mouse Cluster 7 were restricted to high expression levels specifically at the early two‐cell stage, suggesting their identity as minor ZGA genes, analogous to those observed in Human Cluster 2. We observed that a larger proportion of ZGA genes tended to be transcribed de novo in zygotes rather than being maternally inherited and expressed (such as Mouse Cluster 8). This pattern was observed across all five species examined (Figure 1b,c). In addition, we performed KEGG enrichment analysis to further explore the regulatory pathways and functions of ZGA genes. The results showed that ZGA genes in humans, mice and pigs were closely associated with disease development and were primarily enriched in two key pathways: Coronavirus disease (COVID‐19) and Huntington disease. In contrast, regulatory pathways in bovines and goats were essentially the same, focusing on cell signalling and immune regulation. The Neuroactive ligand–receptor interaction and calcium signalling pathway, co‐regulated by *HTR2A*, *TACR3*, *LHCGR* and *PTGER3*, along with the cytokine–cytokine receptor interaction and cell adhesion molecules pathways regulated by *CD40LG*, exhibited the most prominent enrichment (Figure S1b).

It is worth noting that the ZGA gene sets of different species all contain a certain number of noncoding RNAs (ncRNAs), among which the proportion in humans (26%) and pigs (24%) is relatively higher. This observation is consistent with ncRNAs acting as key drivers of epigenetic reprogramming and embryonic developmental programmes during the ZGA stage (Figure 1d) [46, 47, 48, 49, 50]. Intriguingly, the GC content of ZGA genes varies among species, with bovines and goats exhibiting a remarkably higher GC percentage compared to other species (Figure 1e). The GC% density profiles of humans, pigs, bovines and goats exhibited a bimodal distribution, with Peak 1 centered around 40% and Peak 2 predominantly at 52% (45% in goats). In contrast, the GC% density curve for mice displayed only a single peak, with the peak approximately at 46% (Figure 1f). Based on the fact that GC linkages have three hydrogen bonds, high DNA GC content makes a difference in forming DNA secondary structure that further affects the chromatin openness. GC‐rich genes are reported to be associated with the expression of ubiquitous ‘house‐keeping genes’ and highly regulated key developmental genes, with significantly lower methylation levels and high enrichment of H3K4me3 [51]. RNA Pol II independently recognises GC‐rich promoter DNA sequences during ZGA [52]. Concurrently, Dppa2/4 also activate some signalling pathways associated with developmental reprogramming to facilitate ZGA by binding to CG‐rich regions [53].

### 2.2 Sequence Conservation of Cross‐Species Homologous ZGA Genes

**FIGURE 2:**
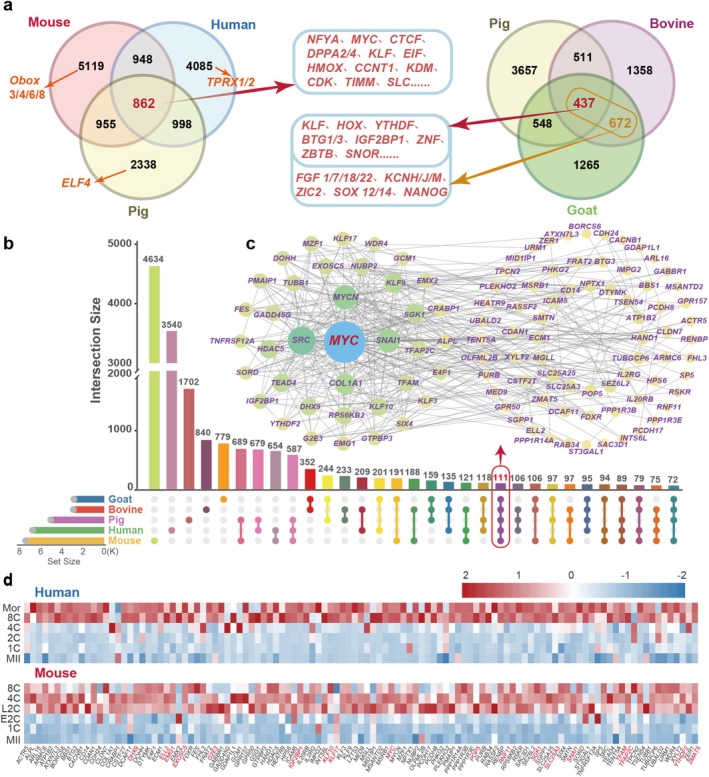
Comparative analysis of homologous ZGA genes across species. (a) Venn diagram showing the overlapping numbers of ZGA genes in mice, humans and pigs, and those in pigs, bovines and goats. (b) UpSet plot depicting the numbers of overlapping ZGA genes at different intersections among the five species. (c) PPI network of homologous genes across species. (d) Heatmaps showing the gene expression levels of 111 homologous genes in mice and humans, with the top 18 genes exhibiting high expression levels indicated in red.

Generally, different genes play unique roles in biological evolution, while the same genes play similar functions and are conserved [54]. We focused our attention on cross‐species conserved ZGA genes. The results of the comparative analysis showed that 862 ZGA genes were shared among the three widely studied model organisms: mice, humans and pigs, while 437 ZGA genes were conserved among pigs, bovines and goats (Figure 2a,b). However, only 111 ZGA genes were commonly found in five species, with *MYC* occupying a central position in the gene interaction network (Figure 2b,c). We attributed this limited overlap to the relatively incomplete genome annotation information for bovines and goats, resulting in only 2977 and 2921 ZGA genes annotated, respectively. Upon close examination of the transcriptional dynamics of those 111 shared genes, it became evident that nearly all of them had exhibited a highly expressed state during the major ZGA in each species (Figure 2d and Figure S2a).

**FIGURE 3:**
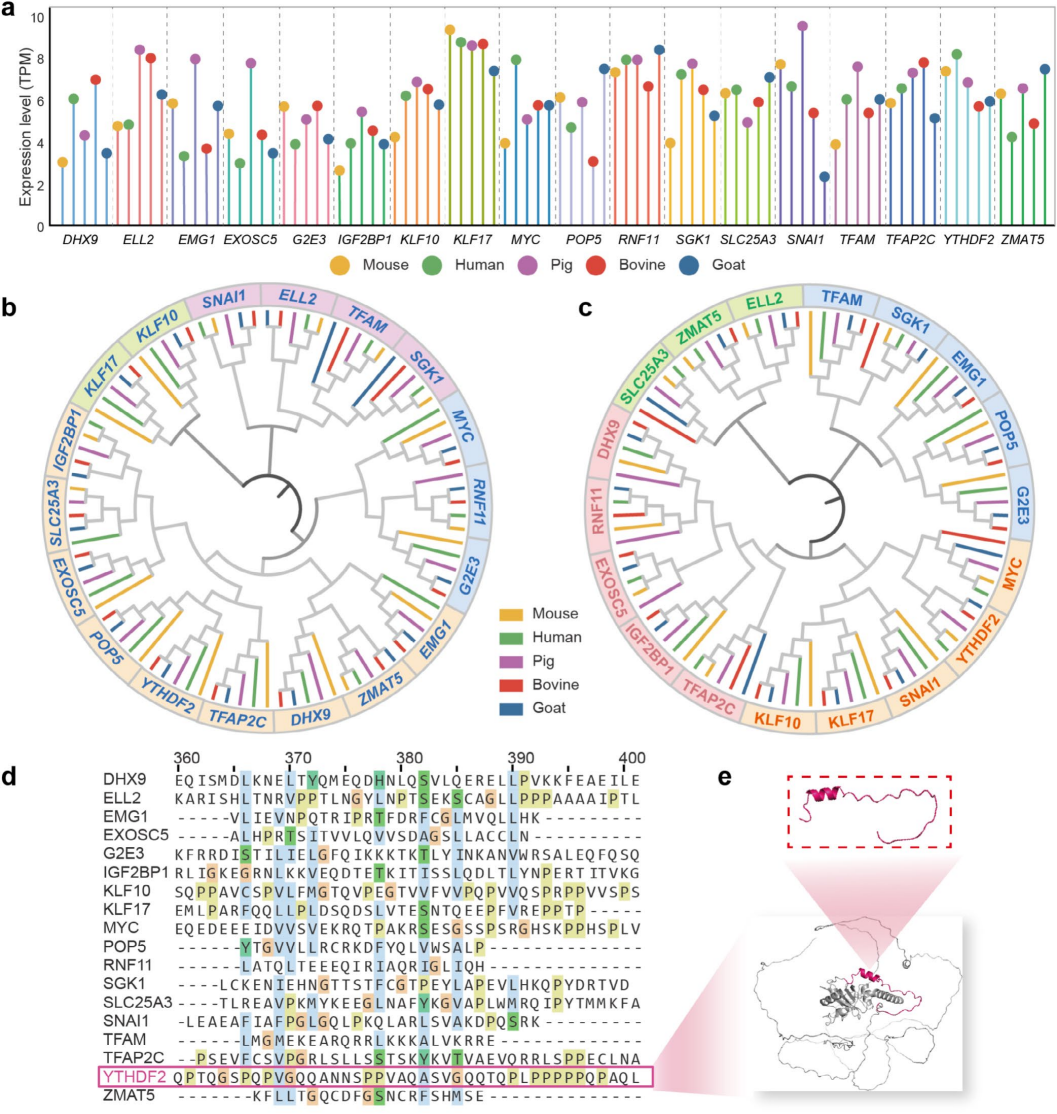
Evolutionary relationships of homologous ZGA genes. (a) Transcript levels of 18 highly expressed genes (TPM > 2) in different species during the major ZGA period. Mice, 2‐cell. Humans and pigs, 8‐cell. Bovines and goats, 16‐cell. (b, c) Phylogenetic tree of homologous genes and proteins across species. (d) Protein sequence alignment showing conserved regions. Colour markers indicate relatively conserved regions, which are coloured by clusters. (e) Functional conserved region structure of YTHDF2.

To further investigate the conserved regions of homologous genes, we selected the top 18 highly expressed ZGA genes for a detailed analysis of their coding sequences (CDS) and amino acid sequences (Figure 3a). The construction of the phylogenetic tree indicated the conservation of orthologs across the five species, with evolutionary divergence primarily observed in paralogs. Consequently, humans were chosen as the representative species for subsequent analyses (Figure 3b,c). Intriguingly, the alignment analysis of protein sequences revealed a highly conserved region positioned between Positions 360 and 400, which also emerges as a potential functional domain (Figure 3d). Taking YTHDF2 as an example, Positions 368–382 are rich in polar residues, while the conserved region from Positions 385 to 400 binds to mRNAs containing multiple m6A‐modified residues (Figure 3e) [55, 56]. YTHDF2 is involved in the degradation of m6A‐containing mRNAs in mammalian gametes [57]. Likewise, a prominently gene‐conserved region was also found in the range of 1496–1585, which was densely enriched with short DNA sequences such as CTGCAG (Figure S2b).

In summary, ZGA genes exhibit high conservation in various species, particularly in specific regions of protein and gene sequences related to function, indicating apparent sequence preferences. The enrichment of short DNA sequence patterns in conserved regions of the gene may be related to gene regulation or other important biological processes.

### 2.3 Interpretable Machine Learning Models Based on Sequence‐Dependent Determinants

The precise triggering of ZGA relies on intricately coordinated mechanisms, in which sequence‐specific binding TFs play a central role [7, 58, 59]. However, how sequences evolve to regulate genes across species during ZGA remains poorly understood. To seek out the regulatory TFs and potential motifs associated with ZGA genes, we extracted the regions surrounding the transcription start sites (TSS −2 and +1 kb) and transcription end sites (TES −1 and +2 kb) of ZGA genes from five species as input sequences for de novo motif analysis. Binding motifs for TFs such as MYC, OTX2, MYOD, POU5F1 and CTCF, and members of the KLF family were identified in both mouse and human sequences, along with motifs for pioneer transcription factors (OBOX, NR5A2, TPRX1/2) known to regulate ZGA (Figure S3) [13, 14, 16]. Due to the limited availability of comprehensive motif databases for pigs, bovines and goats, we utilised existing data to predict potential motifs and their corresponding binding TFs. Interestingly, the mouse ZGA regulator NR5A2 motif was also enriched in the ZGA gene sequences of pigs, bovines and goats (Figure S3). This finding supports the speculation of Johanna Gassler et al. that ‘the regulation of ZGA by orphan nuclear receptors such as Nr5a2 is a conserved mechanism, at least among mammals’ [13, 60, 61]. The above results suggest that mammalian ZGA genes may possess potential unique genetic coding characteristics.

**FIGURE 4:**
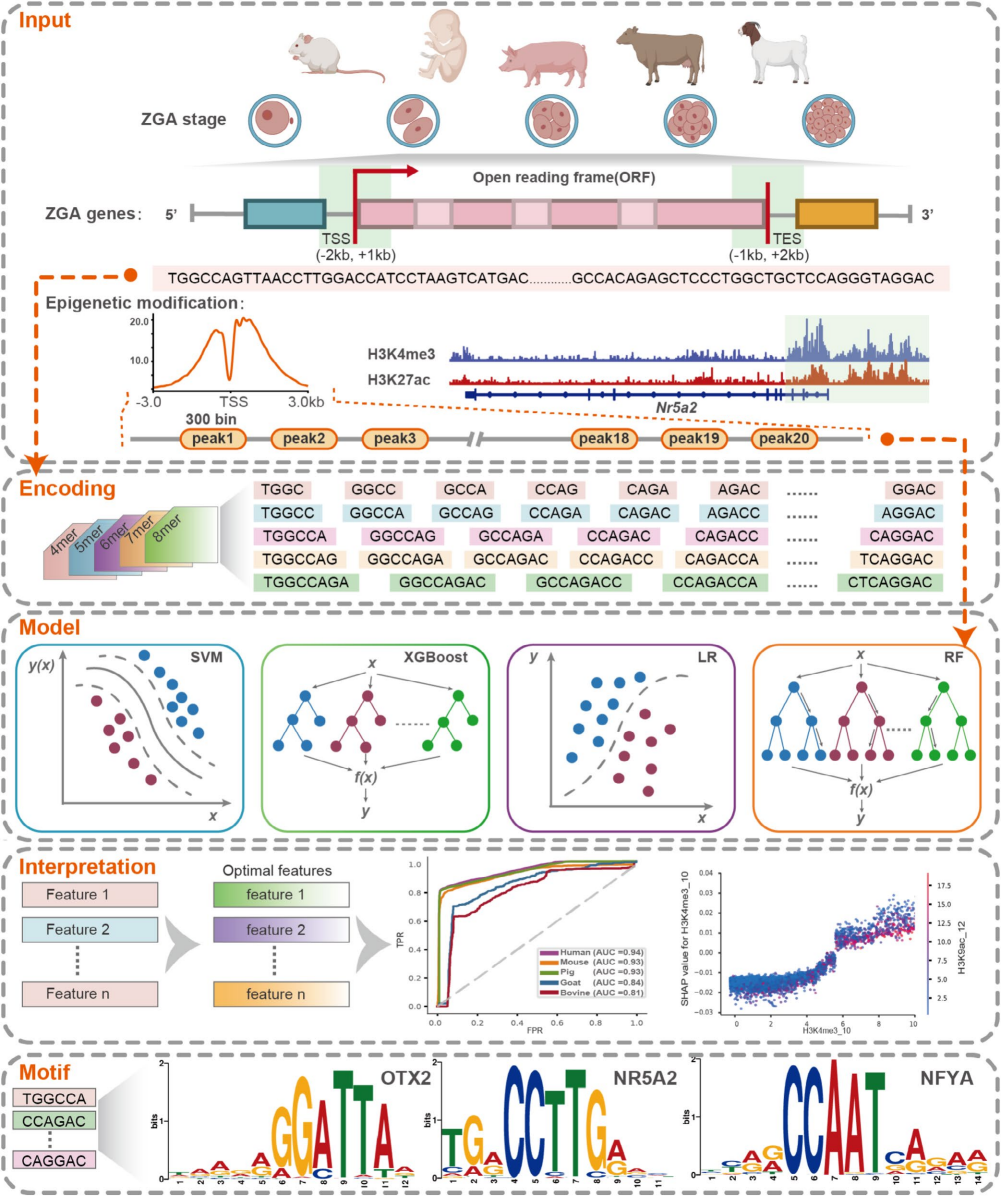
Schematic representation of the interpretable machine learning model for ZGA genes.

**FIGURE 5:**
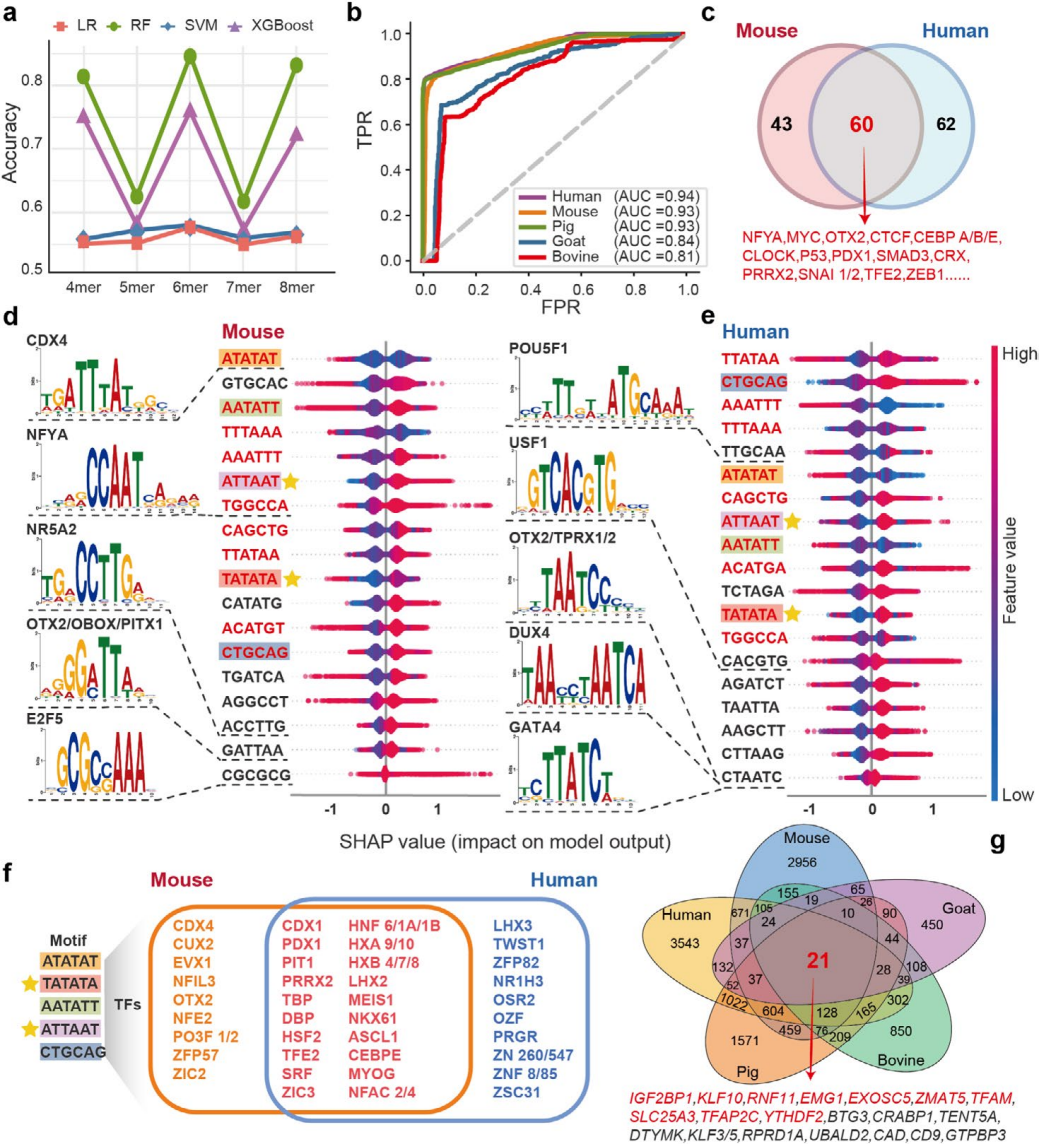
The 6‐mer binding profiles of ZGA gene sequence preferences. (a) Mouse model prediction accuracy under different machine learning algorithms and *k*‐mer combinations. (b) ROC curves of ZGA gene sequences in different species using the RF algorithm. (c) Venn plot showing the overlapping number of TFs that match the features of both mouse and human models. (d, e) Feature contributions of mouse and human models and the TFs matched by partial features. The shared features between human and mouse models are labelled in red, and the shared features among the five models are labelled with background. The common favourable features between models are highlighted by stars. (f) TFs matching to the shared model features across species. (g) Venn plot showing the overlap of genes with genome‐wide model scores above 0.6 across five species.

Next, we asked whether hidden relationships and patterns existed between ZGA genes and sequence features. We employed four distinct machine learning‐based algorithms to predict ZGA genes based on the same input sequences as described above. To enhance the representation of sequence information, we adopted the widely utilised *k*‐mer method as our feature extraction methodology, ensuring a more comprehensive portrayal of the genetic sequences involved (Figure 4) [35]. Insights from previous research were leveraged to guide the selection of parameter *k*, and a range of *k* values from 4 to 8 was adopted in this study [33, 62, 63, 64]. Initially, leveraging the mouse dataset as a starting point, we embarked on a quest to identify the optimal *k* value and the best‐performing model. After a comprehensive evaluation of all combined models, it became evident that the RF‐6mer model outperformed the rest of the models, demonstrating superior performance. Although the RF‐8mer model also achieved impressive results, its longer computation time led us to ultimately adopt the RF‐6mer model as the unified modelling approach for modelling (Figure 5a). The final selection of the *k* value also aligns with the prevailing practice employed in many machine learning approaches based on *k*‐mer, underscoring the reliability of our methodological choice [32, 34, 65, 66]. Then, we applied the RF‐6mer framework to datasets from each of the five species, incorporating grid search and 10‐fold cross‐validation strategies to identify the optimal parameters for each model. Of note, all models exhibited excellent performance, with the human model achieving the highest AUC of 0.94, while the bovine model showed the lowest AUC of 0.81 (Figure 5b).

We used Shapley's feature attribution framework analysis to systematically assess the features that contributed to the model's predictions (see Section 4). By calculating the average absolute value of the SHAP value for each feature across all samples and ranking them, we derived a measure of feature importance for the model. When a high feature value corresponds to a high or low SHAP value, it indicates that the feature acts as a favourable or unfavourable predictor, respectively, for identifying ZGA genes. SHAP analysis showed that over half of the identified features were shared between mouse and human models (Figure 5d,e). Notably, five features (ATATAT, TATATA, ATTAAT, AATATT and CTGCAG) were consistently observed across all species models, accounting for approximately 25% of the total modelled features. The ‘CTGCAG’, which is highly enriched in the alignment profiles of conserved human ZGA gene CDS sequences, was also present in the model features as a pivotal favourable predictor (Figure 5e and Figure S2b). Interestingly, TATATA and ATTAAT were found to be significantly favourable features across models of five species. In other words, the ZGA gene input sequences of mice, humans, pigs, bovines and goats demonstrated a distinct preference for TATATA and ATTAAT sequences. These shared sequence features played a crucial role in the ZGA gene prediction models, suggesting their conservation across the five mammalian species (Figure 5d,e and Figure S4).

To further investigate potential binding TFs, the Tomtom tool was employed to match all sequence features against the mouse and human motif databases. The results showed that 103 and 122 TFs were associated with the mice and human sequence features, respectively, with 60 TFs shared between the two species, including NFYA, MYC and OTX2 (Figure 5c). Importantly, two groups of TFs, namely NR5A2, NFYA and OBOX in mice, and TPRX1/2 and DUX4 in humans, were prominently represented among the matching TFs, which further validates the reliability of our modelling algorithm and feature selection process [13, 14, 16, 43, 44]. Additionally, a valuable set of TFs was also identified on the five shared sequence features, suggesting their important contribution to the regulation of early embryonic development in both mice and humans, with potential implications that could be extrapolated to pigs, bovines, and goats (Figure 5f). It has been reported that TBP might be a cellular memory marker for the maintenance of genome‐wide reprogramming during embryo development, and MEIS1 is closely related to embryo implantation in mice [67, 68]. Yet, two groups of differential TFs were enriched for the same motif. While this might appear trivial, it actually highlights the differences in motif usage and TF functions between humans and mice. In summary, our interpretable machine learning model accurately predicts mammalian ZGA genes, decoding the DNA coding of unique sequence preferences and uncovering the conserved 6‐mer features and potential binding TFs associated with ZGA regulation.

### 2.4 Explainable Model Insights Into Epigenetic Modification Patterns

Many studies have highlighted that multiple types of histone modifications coordinately regulate ZGA in mammals [20, 24, 69]. Consequently, we posit that ZGA genes may exhibit unique histone modification characteristics. To substantiate our hypothesis, we collected ChIP‐seq data of Pol II and 10 histone modifications from mouse two‐cell embryos. Using the signal intensities within the 3 kb flanking regions of the ZGA gene TSS as the input, we developed an interpretable RF model for predicting ZGA genes (Figure 4).

**FIGURE 6:**
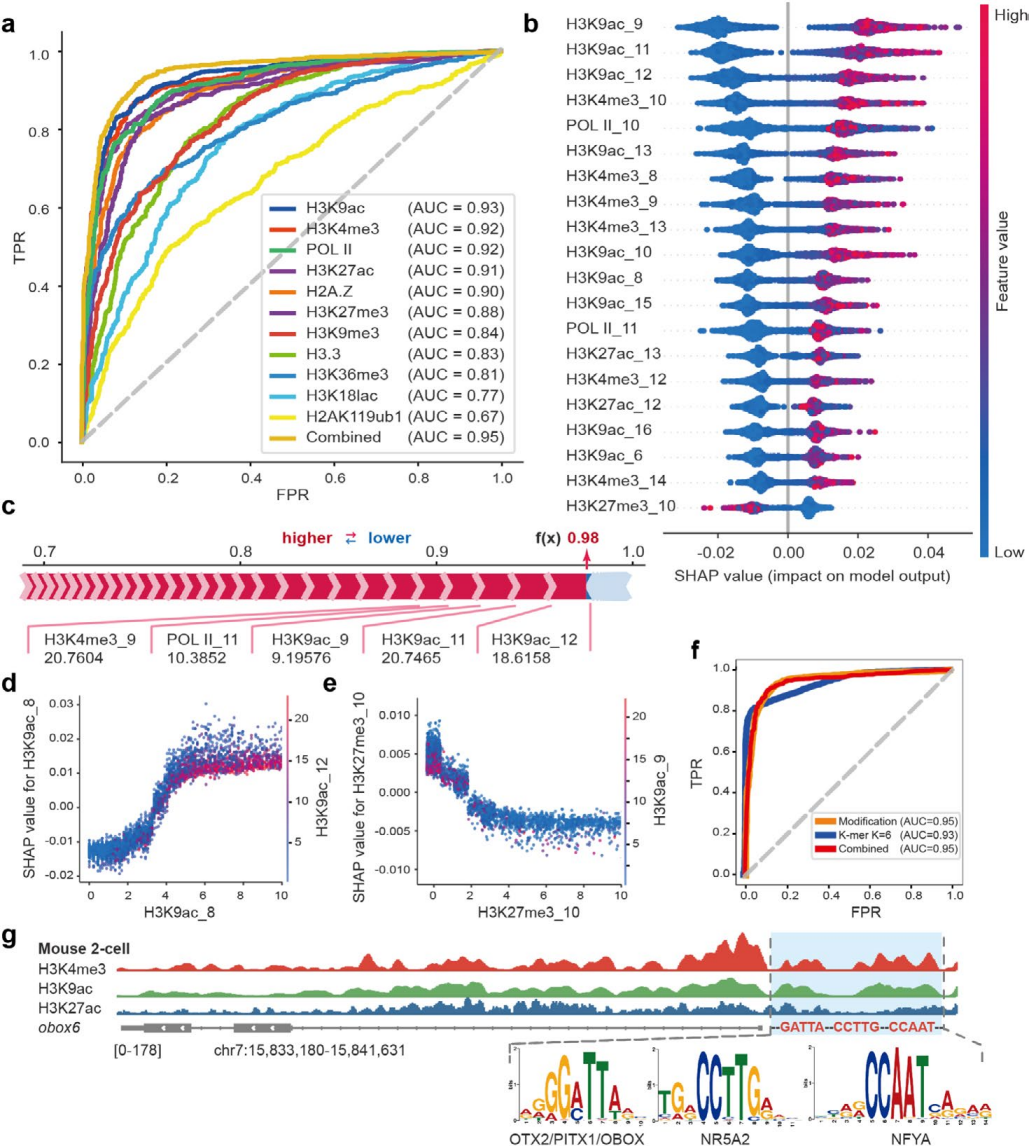
The regulatory syntax on epigenetic modifications of mouse ZGA genes. (a) ROC curves for different epigenetic modifications using the RF algorithm. (b) Feature contributions in the combined model. (c) Explanation of the model prediction results for a sample. (d, e) The scatter plot showing the relationship between the feature values and SHAP values as well as the interaction effects between the features. (f) ROC curves of different models. (g) Schematic representation of the motif features and modification patterns on *obox6* in mouse 2‐cell.

The results showed that the AUC values of the single data prediction results exceeded 0.81, except for H3K18lac and H3K119ub1. After merging all datasets, the overall predictive performance of the model reached an AUC of 0.95 (Figure 6a). Moreover, SHAP analysis showed that features of the H3K9ac, H3K4me3 and Pol II groups almost occupied the top 20 positions in the model, all making favourable contributions to the prediction results. Remarkably, H3K27me3_10 emerged as a distinctively unfavourable feature at the 20th position (Figure 6b). Subsequently, we asked how the intensity of histone modifications affects ZGA gene prediction and the interactions between histone modifications. There was a prominent positive correlation between the signal intensity values of H3K9ac_8 and the corresponding SHAP value, whereas H3K27me3_10 showed a negative correlation. Specifically, upon exceeding the threshold of 4 for the feature value of H3K9ac_8, the SHAP value escalates in tandem with the increasing signal intensity value, thereby enhancing its substantial contribution to the prediction of ZGA genes (Figure 6d). Conversely, when the signal intensity value of H3K27me3_10 surpassed a threshold of 2, the SHAP value decreased as the feature value increased, which aligns with the known inhibitory role of H3K27me3 modification on gene expression (Figure 6e). Interestingly, H3K9ac modification consistently demonstrated strong interactions with H3K9ac_8 and H3K27me3_10 features, further underscoring the significant impact of H3K9ac on ZGA genes (Figure 6d,e). Additionally, a meticulous examination of the genomic locations of all modification features revealed a compelling trend: features with heightened importance tended to localise within 900 bp on the TSS flank of the ZGA gene. Using a sample that had been predicted with high confidence to be a ZGA gene as an example, the top five important favourable features were: H3K9ac_12, H3K9ac_11, H3K9ac_9, POL II_11 and H3K4me3_9 (Figure 6c).

The aforementioned findings showed the distinctive histone modification regulatory syntax exhibited by ZGA genes. Specifically, the region near the TSS of the mouse ZGA genes tends to be more enriched with H3K9ac and H3K4me3 activating histone modifications, while there is less deposition of H3K27me3 repressive modification.

### 2.5 ZGAExplorer Webserver

**FIGURE 7:**
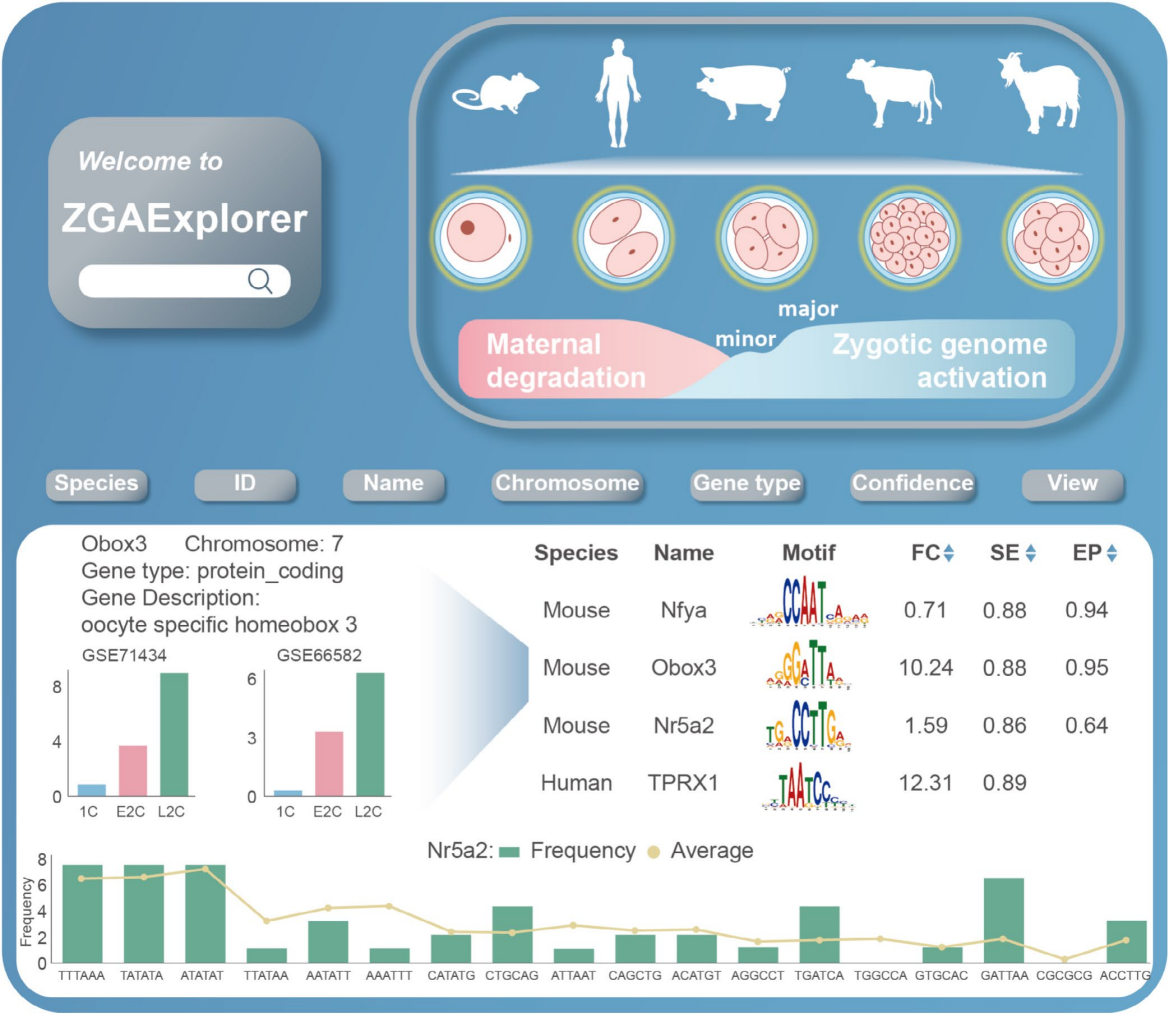
Functions and data visualisation of the ZGAExplorer platform.

Subsequently, to facilitate the identification and analysis of ZGA genes, we developed ZGAExplorer (http://bioinfor.imu.edu.cn/zgaexplorer/index.html), which is a free and user‐friendly web service platform (Figure 7). ZGAExplorer is the most comprehensive ZGA gene information platform to date, integrating approximately 170,000 genes spanning 40 gene types from 17 datasets, encompassing five mammalian species. Detailed information, including species, Ensembl IDs, gene names, chromosomal locations, gene types and motifs, is clearly presented. Simultaneously, ZGAExplorer is an interactive resource platform that provides comprehensive gene annotations and functional insights. Users can perform flexible customised searches and analyses, and visualise gene expression dynamics across different developmental stages.

Importantly, researchers can efficiently conduct rapid screening and identification of ZGA genes by utilising the predicted confidence scores from sequence models and epigenetic modification models, along with fold‐change data in gene expression. The frequency distribution of gene features is also clearly presented. High‐confidence criteria include a sequence model score ≥ 0.6, an epigenetic model score ≥ 0.6 and a gene expression fold change ≥ 2. For a gene in a given species, if its sequence model score is ≥ 0.6 and its expression fold change is ≥ 2 in at least two datasets, it is considered a high‐confidence ZGA gene. For mouse genes, all three conditions must be met. In summary, ZGAExplorer is a comprehensive and user‐friendly platform that facilitates the identification, analysis and visualisation of ZGA genes, providing invaluable resources for advancing related experimental research.

## 3 Discussion

The functional genes undergoing continuous genetic evolution across diverse species play pivotal roles in preimplantation embryonic development and zygotic genome activation. However, how mammalian ZGA is regulated, which genes are involved, and how conserved the genes are across species remains poorly understood [4]. Here, we conducted a systematic reanalysis of RNA‐seq data from oocytes and preimplantation embryos and integrated previous research findings on ZGA genes to compile a relatively complete and reliable set of ZGA genes for mice, humans, pigs, bovines and goats. This endeavour has revealed 111 homologous genes, suggesting their evolutionary conservation and importance for zygotic genome activation. We discovered potential conserved regions within homologous ZGA genes utilising gene and protein sequence alignment and phylogenetic analyses. Moreover, by conducting de novo motif analysis, we speculated on the potential binding TFs of ZGA genes for pigs, bovines and goats, including experimentally demonstrated KLF4 and ELF4 [70, 71].

To explore the key elements influencing the expression of ZGA genes, we evaluated four different machine learning‐based methods for predicting ZGA genes based on DNA sequence and histone modification data. The RF model outperformed the corresponding models of several other methods. First, five decisive six‐base features consistently emerged as pivotal predictors in the models across five species, highlighting the conserved evolutionary patterns within mammalian ZGA gene sequences. By aligning these salient sequence features with TF motif databases, we obtained a set of reliable TFs, implying their interactions with the signature sequences and pivotal regulatory roles during mammalian ZGA. We speculated that the model feature sequences may play a promoter‐ or enhancer‐like function, providing robustness for the initiation of ZGA through the binding of TFs. The distinctive sequence preference of the ZGA gene may contribute to the strong selective pressure on their amino acid sequences, as well as to the biological specificity in target genes and diversity in function among proteins. Moreover, the histone modification model indicated that regions near the TSS of mouse ZGA genes are typically enriched with activating histone modifications such as H3K9ac and H3K4me3, while displaying a reduced abundance of the repressive modification H3K27me3. This underscores the role of histone modifications in the coordinated regulation of ZGA. When integrating both types of data to construct a ZGA gene prediction model, the performance reached an AUC of 0.95, further demonstrating that both genetic sequence features and epigenetic modifications contribute significantly to the regulation of ZGA genes (Figure 6f,g).

Subsequently, by employing the well‐trained ZGA gene prediction model, we conducted a comprehensive screening for the reference genomes of five mammals and generated a detailed report on the prediction confidence levels of ZGA genes (Table S2). Remarkably, among the genes exhibiting high confidence scores (confidence > 0.6), a set of 21 shared genes was identified across the five species. Ten genes, including *YTHDF2* and *IGF2BP1*, were found to be in concordance with the highly expressed homologous ZGA genes (Figures 3a and 5g). The cross‐species average probability scores of *SLC25A3* and *TFAP2C* surpassed 0.9, thereby emphasising their prominent significance and the requisite attention they deserve. We postulate that these genes hold significant implications for the mammalian ZGA. Finally, we integrated multiple transcriptomic datasets and calculated the fold changes in differential gene expression during the ZGA stage (Table S2), aiming to provide additional references for the identification of ZGA genes.

To facilitate the identification and analysis of ZGA genes, we developed ZGAExplorer, a free and user‐friendly web service platform. ZGAExplorer is not only the most comprehensive ZGA gene information platform to date, integrating 17 datasets from five mammals, covering approximately 170,000 genes of 40 gene types, but also provides detailed interactive resources, including species, Ensembl ID, gene name, chromosomal location and gene type. Users can rapidly screen and identify ZGA genes by referring to the prediction confidence scores of the reference sequence model and the epigenetic modification model as well as the fold change data of gene expression.

In summary, through multi‐omics data integration, we have compiled a comprehensive and reliable multi‐species ZGA gene set, shedding light on the remarkable evolutionary disparities and underlying conservation among diverse species. More interestingly, an interpretable machine learning model based on sequence‐dependent determinants showed excellent performance in predicting ZGA genes, uncovering conserved motif features and potential binding TFs among mammals. The results demonstrated that both genetic sequence features and epigenetic modification features are equally crucial for the regulation of ZGA genes. ZGAExplorer also provided a visualisation platform to support further research on ZGA genes. Our discoveries offer valuable insights into the study of ZGA regulators in alternative animal models, contributing to the design of artificial ZGA regulatory elements and overcoming embryonic developmental arrest.

## 4 Methods

### 4.1 Dataset Collection

Publicly available histone modification ChIP‐seq data for mouse 2‐cell were downloaded from the NCBI Gene Expression Omnibus (GEO) database, and the GSE IDs are as follows:

H3K4me3 (GSE71434), H3K9me3 (GSE97778), H3K27ac (GSE185653), H3K27me3 (GSE73952), H2AK119ub1 (GSE153531), H2A.Z (GSE188588), H3K36me3 (GSE112835), H3K18lac (GSE234027), H3K9ac (GSE143523), H3.3 (GSE139527) and Pol II (GSE135457).

Publicly available RNA‐seq data of mice, humans, bovines and goat oocytes and embryos were also obtained from the GEO database with the following GSE IDs: mice (GSE71434; GSE211845; GSE66582; GSE213407; GSE169632), humans (GSE36552; GSE101571; GSE44183; GSE71318), pigs (GSE139512; GSE164812), bovines (GSE52415; GSE59186) and goats (GSE129742). The pig RNA‐seq data were downloaded from the Genome Sequence Archive (GSA) database with accession number CRA004237 and CRA006174. The goat RNA‐seq data were downloaded from the SRA database with accession number PRJNA543590.

### 4.2 RNA‐seq Analysis

Trimmed RNA‐seq reads were aligned to the respective species' genomes employing Hisat2 [72] (version 2.2.0) with default settings. Sam files were converted to bam format using Samtools (version 1.9). Gene expression levels were quantified and normalised to TPM (transcripts per million) utilising the featureCounts [73] (version 1.6.2). Differential gene expression analysis was conducted by R package DEseq2 [74], identifying genes as differentially expressed if they exhibited a fold change > 2 and adjusted *p* < 0.05.

### 4.3 Identification and Screening Criteria of ZGA Genes

ZGA genes were defined based on reference RNA‐seq data using staged embryos of five mammalian species. ZGA genes in mouse embryos were defined as the TPM ratios of early 2C/1C > 2.5, late 2C/1C > 3, early 2C/MII > 3, late 2C/MII > 3, or late 2C/early 2C > 3, when the TPM values of MII or 1C < 0.5. In human and pig embryos, ZGA genes were defined as the TPM ratios of 4C/1C > 2.5,8C/1C > 3, 4C/MII > 3, 8C/MII > 3, or 8C/4C > 3, when the TPM values of MII or 1C < 0.5. In bovine and goat embryos, ZGA genes were defined as the TPM ratios of 8C/4C > 2.5, 16C/4C > 3, 8C/MII > 3, 16C/MII > 3, or 16C/8C > 3, when TPM values of MII or 4C < 0.5. In selecting thresholds and identification strategies for ZGA genes, we drew on the standards and methods from previous studies. To ensure the comprehensiveness and accuracy of the final ZGA gene sets for each species, we integrated candidate ZGA genes identified in other studies and incorporated them into our final analysis.

### 4.4 ChIP‐Seq Data Analysis

Fastq format data were first controlled by FastQC and trimmed to remove adapters and low‐quality sequences using Trim‐galore (version 0.6.1) with default parameters. Trimmed reads were then aligned to each species' genomes using bowtie2 [75] (version 2.5.4) with default parameters. Sam files were converted to bam files using Samtools (version 1.9) and duplicate bam files were merged. To quantify the ChIP‐seq intensity and make it comparable, we extracted the region encompassing 3 kb upstream and downstream of the ZGA gene TSS and divided it equally into 20 bins. Read counts were normalised by computing the fragments per kilobase (FPKM) of the exon model per million mapped reads.

### 4.5 Protein Sequence Analysis

The CDS sequences of genes were obtained from GenBank (https://www.ncbi.nlm.nih.gov/genbank/), while the protein sequences were retrieved from UniProt (https://www.uniprot.org/). PDB (https://www.rcsb.org/) and AlphaFold (https://alphafold.com/) were then used to download the protein structures. The preliminary phylogenetic tree was constructed using the maximum likelihood method in MEGA 11.0.10, with the Bootstrap method value set to 500. iTOL (https://itol.embl.de/) was utilised to beautify the phylogenetic tree. PyMol 2.6 was employed for structural comparison and analysis. For result visualisation, iTOL and Jalview were used.

### 4.6 Motif Analysis

Motif discovery of the extracted *k*‐mer features was conducted with the MEME [76] (version 5.5.6). To identify motifs enriched in the ZGA gene sequences of each species, de novo motif enrichment analysis was performed using HOMER (version 5.1), based on the HOMER and JASPAR databases, excluding fly and yeast backgrounds.

### 4.7 Data Visualisation

Data visualisation was performed primarily using R (version 4.2.0), including the R/bioconductor package [77]. The PPI analysis of genes was based on the STRING database [78], which contains known and predicted protein–protein interactions. For species present in the database, we constructed a network using the target gene list. The Integrative Genomics Viewer (IGV) was applied to visualise the genome browser view [79].

### 4.8 Machine Learning Model Building

Non‐ZGA genes in embryos of different species were defined as genes with a sum of TPM values of gene expression < 2 during preimplantation embryo development. We randomly sampled the same number of genes from the non‐ZGA gene set as from the ZGA genes to balance the dataset. From the reference genome of each species, the flanking region sequences of TSS (−2 and +1 kb) and TES (−1 and +2 kb) of ZGA genes and non‐ZGA genes were extracted as positive and negative sample sets. The dataset of each species was randomly split into training and test subsets with the proportions of 80% and 20%, respectively.

We employed the frequency patterns of *k*‐mer (words of length *k*) with a sliding window with a single nucleotide step as the only feature extraction method for gene sequences. For each given nucleotide sequence containing A, C, T, and G, a sliding window of length *k* and Step 1 was used to intercept the sequence. Each subsequence with *k* nucleotides was called a *k*‐mer pattern. For a given *k*, we can get 4^*k*^ *k*‐mer patterns. For example, when *k* = 3, 64 modes can be obtained, such as ‘AAA’, ‘AAC’, ‘AAT’, and so forth. *K*‐mer patterns are a simpler model than TFBS motifs, as they do not limit information representation to sequences that represent known TFBS motifs. We drew on the experience of previous studies in the selection of *k* [33, 62, 63, 64]. In this study, we took *k* = 4–8 and selected the *k* that gave the best model performance.

For comparison, we evaluated four popular machine learning classification algorithms, including SVM, random forest (RF), LR, and extreme gradient boosting (XGBoost). XGBoost was implemented using the xgboost python package (version 2.0.1), and other model algorithms are scikit‐learn.

Extracting sufficient and effective discriminant features is considered to be the most important step in developing an accurate prediction method. To avoid the introduction of redundant or irrelevant information as much as possible, which may lead to over‐fitting issues or reduce the generalisation capacity of the prediction model, we combined the maximal information coefficient (MIC) and feature selection strategies to obtain the optimal feature set. In addition, grid search and tenfold cross‐validation methods were used to search for the best hyperparameters.

### 4.9 Evaluation Metrics

In this study, the following four classic evaluation indicators for binary classification were used to evaluate the performance of the model, namely Sensitivity, Precision, Accuracy, and *F*1 score, which are expressed as follows:

(1) $$\text{Sensitivity}=\frac{\mathrm{TP}}{\mathrm{TP}+\mathrm{FN}}$$

(2) $$\text{Precision}=\frac{\mathrm{TP}}{\mathrm{TP}+\mathrm{FP}}$$

(3) $$\text{Accuracy}=\frac{\mathrm{TP}+\mathrm{TN}}{\mathrm{TP}+\mathrm{TN}+\mathrm{FP}+\mathrm{FN}}$$

(4) $$F1\;\text{score}=\frac{2\mathrm{TP}}{2\mathrm{TP}+\mathrm{FP}+\mathrm{FN}}$$

where TP, TN, FP, and FN represent true positive, true negative, false positive, and false negative samples, respectively. Furthermore, we also used the receiver operating characteristic (ROC) curve to evaluate the performance of the proposed model method. The AUC is a comprehensive indicator of the performance quality of a binary classifier.

### 4.10 Model Interpretation

The SHAP algorithm is a game theoretic approach for explaining the output of machine learning models. The SHAP value quantifies the contribution of each feature to the prediction outcome. We ranked the input features according to the mean absolute SHAP value and displayed the distribution of SHAP values for the entire dataset for each species in our model. The SHAP method was implemented by the shap python package (version 0.45.0).

## Author Contributions

**Yongchun Zuo:** conceptualisation. **Jixiang Xing:** visualisation, formal analysis, writing – original draft. **Siqi Yang:** formal analysis. **Yuchao Liang:** formal analysis. **Pengwei Hu:** data curation, writing – review and editing. **Bingjie Dai:** data curation, writing – review and editing. **Hanshuang Li:** data curation, writing – review and editing. **Yongqiang Xing:** data curation, writing – review and editing.

## Ethics Statement

This article does not contain any experimental studies with human or animal subjects.

## Conflicts of Interest

The authors declare no conflicts of interest.

## Supporting information


**Figure S1.** Expression levels and KEGG pathway analysis of ZGA genes in different species. (a) Transcriptional dynamics and clustering of ZGA genes in fertilised embryos of pigs, bovines and goats. (b) The bubble plot showing the KEGG enrichment pathways of ZGA genes.
**Figure S2.** Expression levels and sequence alignment analysis of homologous genes. a Expression dynamics of homologous genes in pigs, bovines and goats. II, MII. 1, 1‐cell. 2, 2‐cell. 4, 4‐cell. 8, 8‐cell. 16, 16‐cell. M, morula. B, blastocyst. (b) Human gene sequence alignment showing conserved regions. The recurring short meaningful sequence patterns are highlighted in red.
**Figure S3.** Representative motifs and corresponding TFs identified from the de novo motif discovery.
**Figure S4.** Feature contributions of pig, bovine and goat models.

**Table S1.** List of ZGA genes in mouse, human, pig, bovine and goat.

**Table S2.** Confidence scores for multi‐species ZGA gene prediction based on sequence and epigenetic modification models and gene expression fold changes.

## Data Availability

The data that support the findings of this study are available from the corresponding author upon reasonable request.
