# Supplementary material for: Deciphering Sequence Determinants of Zygotic Genome Activation Genes: Insights From Machine Learning and the ZGAExplorer Platform
Source: Cell Prolif. 2025 Apr 18;58(12):e70039. doi: 10.1111/cpr.70039 (PMC12686121; doi:10.1111/cpr.70039)
Supplement: Supplementary file 1 — Figure S1. Expression levels and KEGG pathway analysis of ZGA genes in different species. (a) Transcriptional dynamics and clustering of ZGA genes in fertilised embryos of pigs, bovines and goats. (b) The bubble plot showing the KEGG enrichment pathways of ZGA genes. Figure S2. Expression levels and sequence alignment analysis of homologous genes. a Expression dynamics of homologous genes in pigs, bovines and goats. II, MII. 1, 1‐cell. 2, 2‐cell. 4, 4‐cell. 8, 8‐cell. 16, 16‐cell. M, morula. B, blastocyst. (b) Human gene sequence alignment showing conserved regions. The recurring short meaningful sequence patterns are highlighted in red. Figure S3. Representative motifs and corresponding TFs identified from the de novo motif discovery. Figure S4. Feature contributions of pig, bovine and goat models. [file CPR-58-e70039-s002.docx]

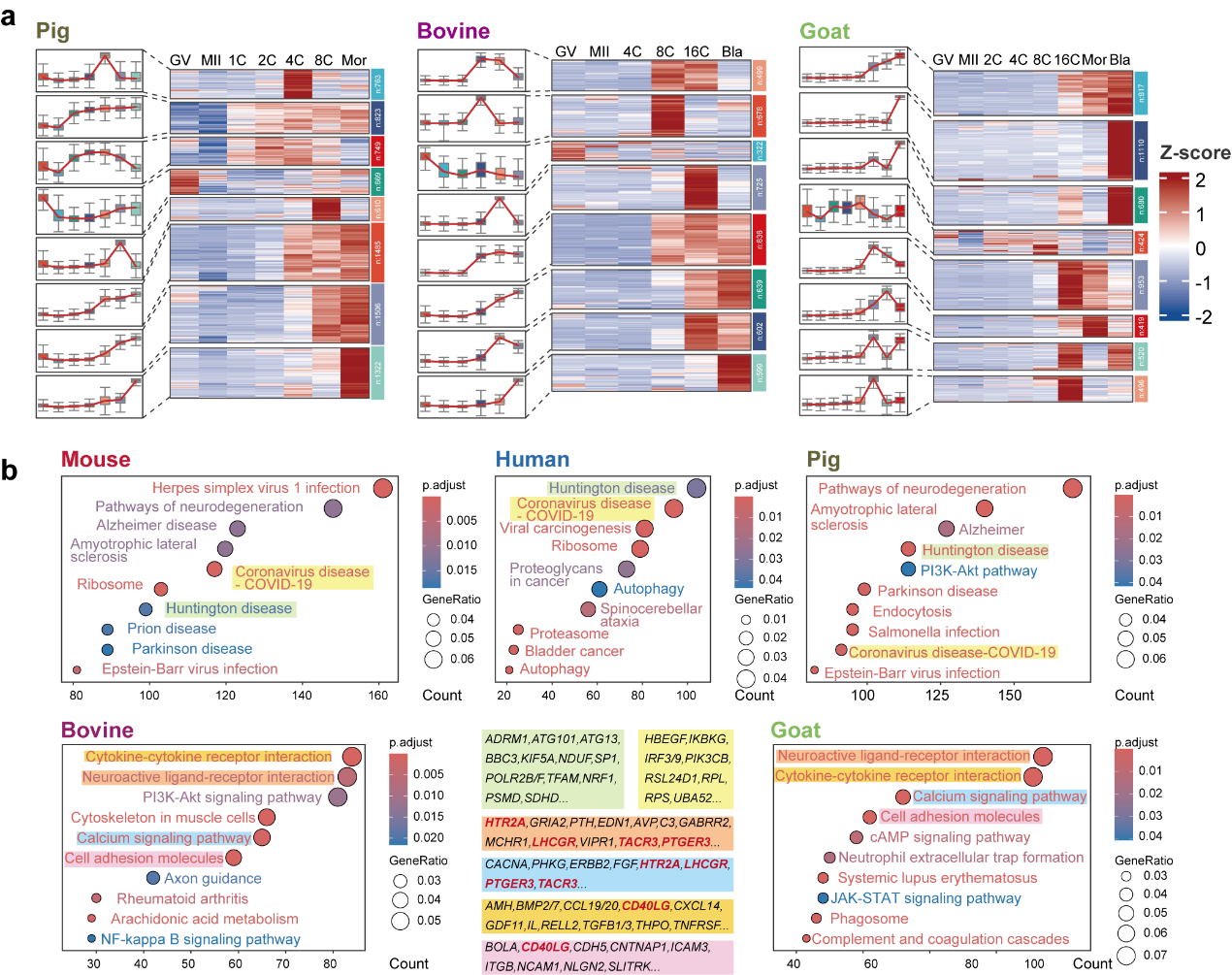
 **Figure S1.** **Expression levels and KEGG pathway analysis of ZGA genes in different species. a** Transcriptional dynamics and clustering of ZGA genes in fertilized embryos of pigs, bovines, and goats. **b** The bubble plot showing the KEGG enrichment pathways of ZGA genes.


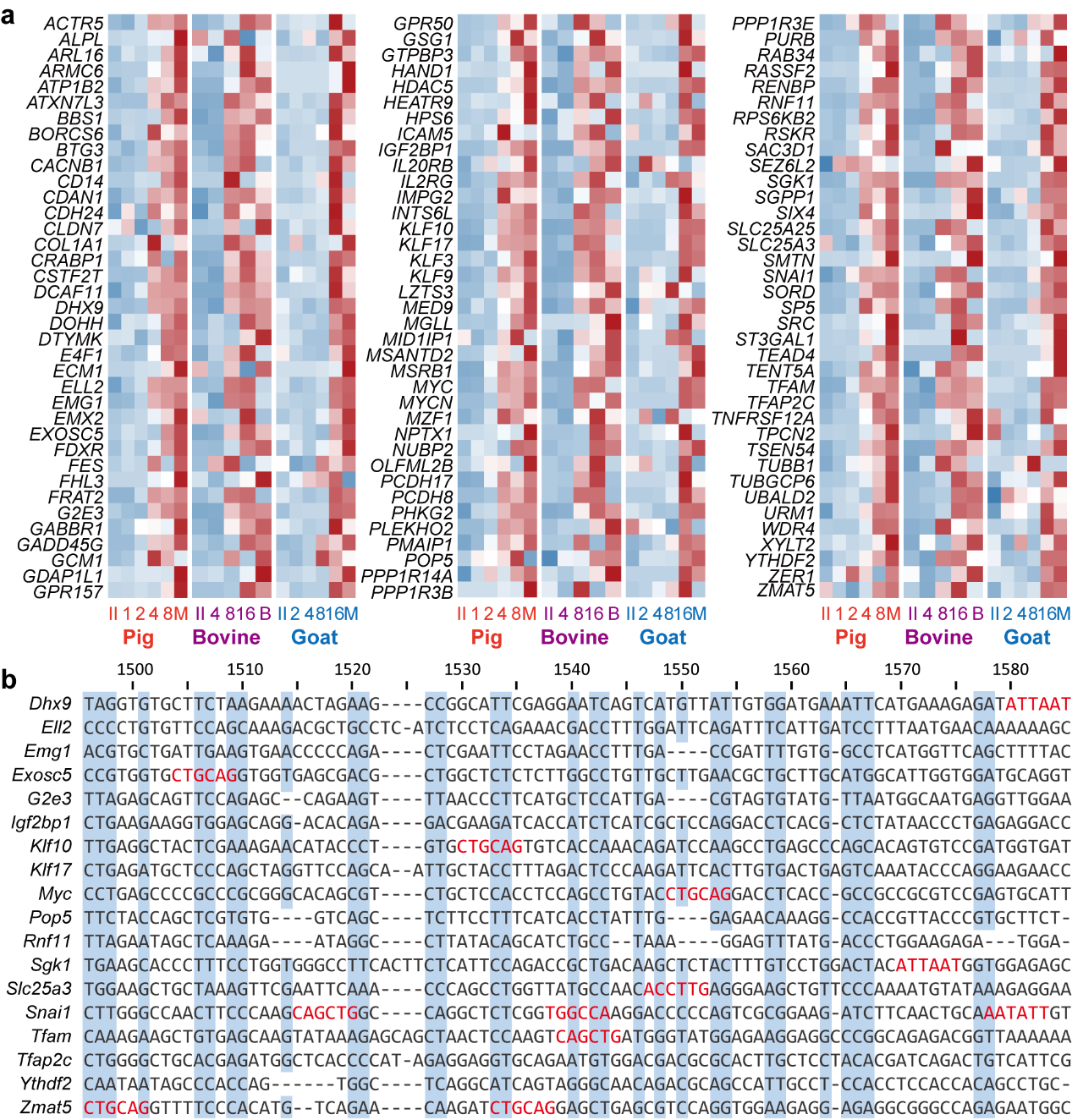


**Figure S2. Expression levels and sequence alignment analysis of homologous genes. a** Expression dynamics of homologous genes in pigs, bovines, and goats. II, MII. 1, 1-cell. 2, 2-cell. 4, 4-cell. 8,8-cell. 16,16-cell. M, morula. B, blastocyst. **b** Human gene sequence alignment showing conserved regions. The recurring short meaningful sequence patterns are highlighted in red.


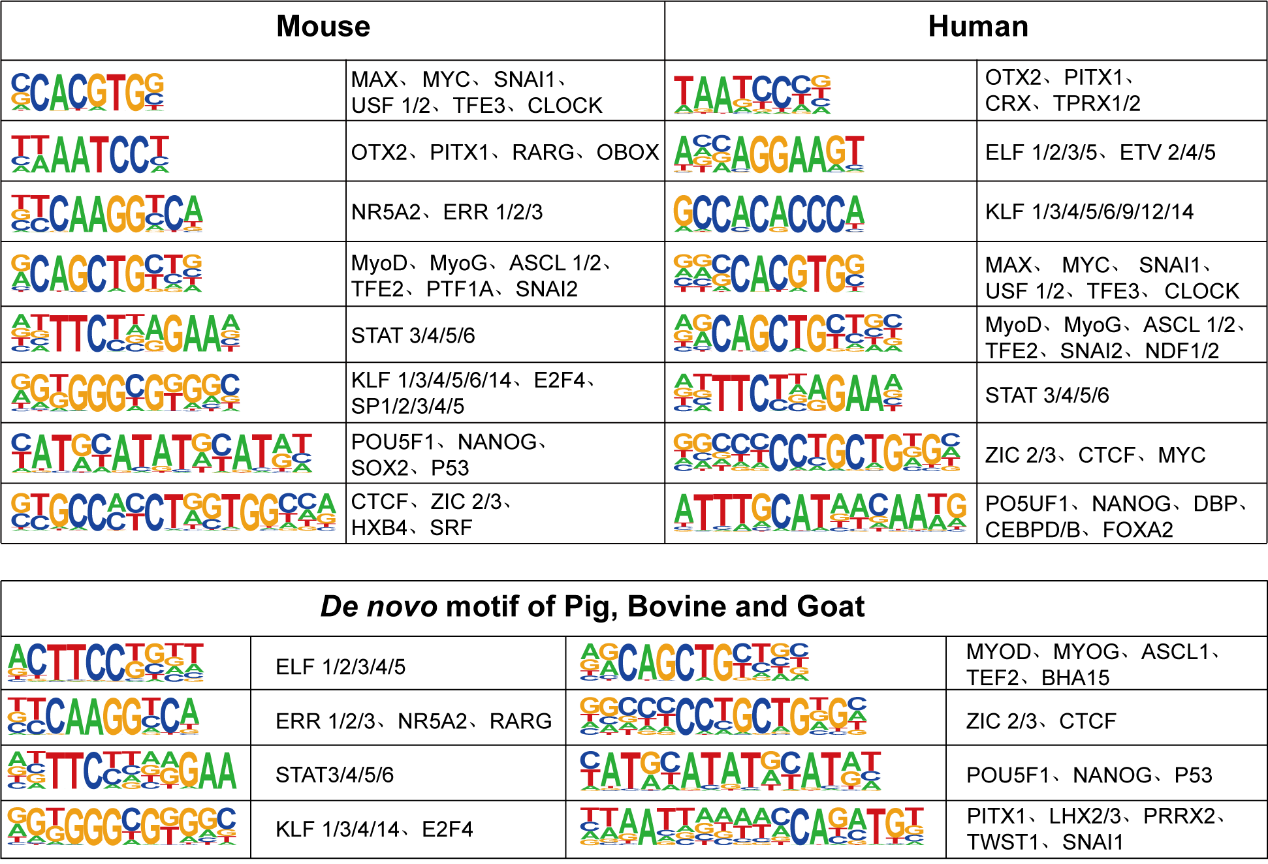


**Figure S3. Representative motifs and corresponding TFs identified from the de novo motif discovery.**


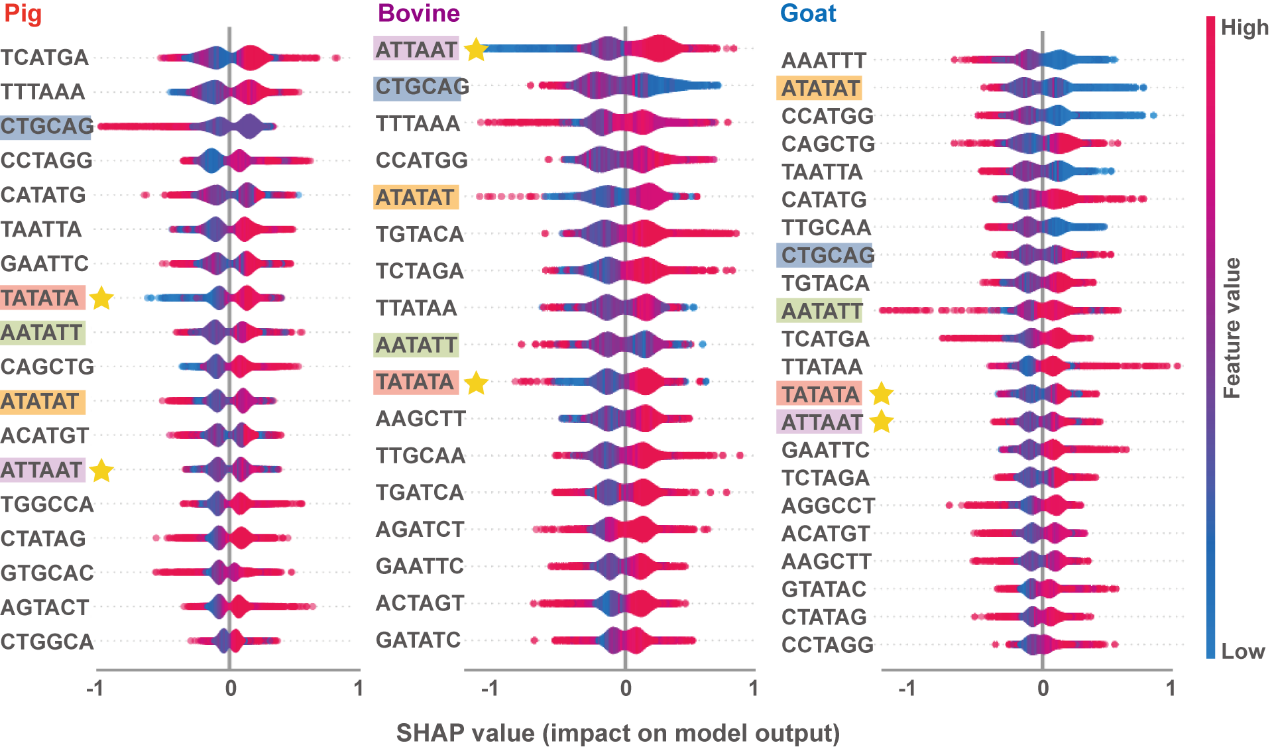


**Figure S4. Feature contributions of pig, bovine and goat models.**
